# First-Line Faricimab in Diabetic Macular Edema: Insights from a Real-World Treatment-Naïve Population in Austria

**DOI:** 10.3390/jcm15103747

**Published:** 2026-05-13

**Authors:** Paul Widmann-Sedlnitzky, Kim Lien Huber, Irene Steiner, Heiko Stino, Laura Kunze, Tilman Schmoll, Bianca S. Gerendas, Katharina Kriechbaum, Stefan Sacu, Andreas Pollreisz

**Affiliations:** 1 Department of Ophthalmology, Medical University of Vienna, 1090 Vienna, Austria; paul.widmann-sedlnitzky@meduniwien.ac.at (P.W.-S.);; 2 Institute of Medical Statistics, Center for Medical Data Science, Medical University of Vienna, 1090 Vienna, Austria; 3 Carl Zeiss Meditec AG, 73447 Oberkochen, Germany

**Keywords:** diabetic retinopathy, diabetic macular edema, faricimab, anti-VEGF, treatment-naïve, retrospective studies

## Abstract

**Background**: Diabetic macular edema (DME) is a leading cause of vision loss. Although real-world data on faricimab, a bispecific antibody targeting vascular endothelial growth factor-A and Angiopoietin-2, are expanding, its long-term durability in routine clinical practice has not yet been fully established. We evaluated effectiveness, anatomic response and treatment durability of first-line faricimab in treatment-naïve DME. **Methods**: We conducted a single-center, retrospective cohort study of treatment-naïve DME eyes initiated on intravitreal faricimab (August 2023–October 2024) in a real-world setting. After a loading phase, eyes were managed with a treat-and-extend or pro re nata regimen. The primary endpoint was retreatment interval at 48 weeks. Secondary endpoints were retreatment interval at weeks 12, 24 and 36; change in visual acuity (VA); central subfield thickness (CST); and optical coherence tomography (OCT) fluid. **Results**: Fifty-two eyes from 40 consecutive patients were included (baseline VA 65.96 ± 13.55 letters; CST 426.56 ± 106.72 µm). Mean injections were 4.02 ± 1.11 between months 1–6 and 1.90 ± 0.98 between months 7–12. VA improved by +8.46, +7.57, +7.65 and +7.72 letters at 12, 24, 36, and 48 weeks (all *p* < 0.0001), respectively. Relative CST decreased by −28.05%, −27.01%, −29.46% and −25.22% at the same time points (all *p* < 0.0001). At week 48, 15.4% of eyes were on a treatment interval of less than 12 weeks, 23.1% were between 12 and 16 weeks, and 46.1% were on 16 or more weeks; 15.4% were managed PRN. **Conclusions**: First-line faricimab in treatment-naïve DME in a real-world setting yielded clinically meaningful and durable extensions in treatment intervals, alongside sustained functional and anatomical improvements.

## 1. Introduction

Diabetic macular edema (DME) has become the leading cause of vision impairment related to diabetic retinopathy in developed countries [1]. DME arises when hyperglycemia and inflammation disrupt the inner blood–retina barrier via angiogenic mediators such as vascular endothelial growth factor-A (VEGF-A) and Angiopoietin-2 (Ang-2). VEGF-A increases vascular permeability, while Ang-2 increases endothelial sensitivity to VEGF-A, collectively promoting fluid leakage into the macula [2].

Intravitreal injections of anti-VEGF agents are currently considered the gold standard for managing DME, having largely replaced traditional focal or grid laser therapies due to superior visual outcomes [3,4]. Despite their effectiveness, regular and frequent injection schedules are required to sustain therapeutic outcomes [5,6,7].

The US Food and Drug Administration and the European Medicines Agency have approved faricimab (Vabysmo; Genentech, San Francisco, CA, USA), a novel bispecific antibody that simultaneously targets and inhibits VEGF-A and Ang-2 in 2022 [8]. This dual inhibition approach, demonstrated in the YOSEMITE and RHINE phase III clinical trials, achieved noninferior visual improvements compared to aflibercept, with dosing intervals potentially extending up to 16 weeks [9]. Nonetheless, outcomes from clinical trials may not fully align with real-world clinical settings due to differences in patient populations and treatment regimens [10].

Real-world evidence has indicated potential advantages of initiating faricimab therapy, including improvements in anatomical parameters and the possibility of extended treatment intervals compared to conventional anti-VEGF treatments [11,12]. However, data specifically concerning treatment-naïve patients initiating faricimab therapy as their primary intervention in everyday clinical practice remains sparse, with most previous studies focusing on short-term follow-up [13,14,15].

This study, therefore, aims to evaluate the efficacy, safety, and visual and anatomical outcomes of treatment-naïve DME patients receiving faricimab as first-line therapy within routine clinical practice, addressing a significant gap in current knowledge and providing insights for individualized patient management.

## 2. Methods

In this retrospective, longitudinal study, we identified patients diagnosed with DME who were initiated on intravitreal faricimab between August 2023 and October 2024 at the Department of Ophthalmology, Medical University of Vienna. Patient data were retrieved from the electronic medical records (VIBES registry). Ethical approval was granted by the ethics committee at the Medical University of Vienna (EK-Nr. 2095/2018), ensuring compliance with the Declaration of Helsinki. Written informed consent was obtained from all participating patients for the inclusion of their data and subsequent analyses.

### 2.1. Inclusion and Exclusion Criteria

Eyes with DME of patients with type 1 or type 2 diabetes mellitus were included in this study if the affected eye had never before received any form of intravitreal treatment. Inclusion criteria required intravitreal treatment initiation with faricimab as a first-line agent. Additional inclusion criteria involved a complete clinical evaluation at baseline and a follow-up time of at least 48 weeks. Patients were excluded if they presented with significant media opacities preventing optical coherence tomography (OCT) imaging, the presence of other retinal diseases or a history of vitreoretinal or cataract surgery within the preceding 6 months.

### 2.2. Clinical Assessment and Imaging

Patient data, including clinical examinations, visual acuity measurements, treatments administered and imaging outcomes, were collected retrospectively from electronic medical records. Macula-centered OCT scans (6 mm × 6 mm) had been performed during each visit using either spectral domain OCTs (Cirrus, Carl Zeiss Meditec, Dublin, CA, USA) or swept-source OCT (Triton, Topcon, Tokyo, Japan). Central subfield thickness (CST) measurements were automated, consistently defined by the internal limiting membrane and the anterior boundary of the retinal pigment epithelium for both imaging systems. Visual acuity (VA) measurements were performed using the Autorefractometer Nidek AR-1 (NIDEK Co., Ltd., Aichi, Japan) with Snellen decimal charts. Partial-line scoring was recorded as additional correctly identified symbols (e.g., p+2). For analysis, Snellen decimal was converted to logMAR, assuming 0.02 logMAR per additional symbol, and approximate early treatment diabetic retinopathy study (ETDRS) equivalent letter scores were derived from the corresponding logMAR values [16].

Faricimab was initiated using monthly loading injections, followed by a treat-and-extend (T&E) or pro re nata (PRN) regimen guided by changes in CST and VA, determined by clinical judgment of the treating ophthalmologist. All injection-related complications and subsequent treatment adjustments were documented at each visit.

### 2.3. Follow-Up Timepoints

Treatment response was evaluated at four predefined time points at weeks 12, 24, 36, and 48. As follow-up visits occurred at varying intervals, the first visit occurring after the respective interval was documented.

### 2.4. Primary and Secondary Outcomes

The primary endpoint was the retreatment interval 48 weeks after starting faricimab. Secondary outcomes were injection intervals at weeks 12, 24, 36 and 48 after treatment initiation, changes in VA, CST, CST-response and presence of any intra- or subretinal fluid at the indicated timepoints. The CST-response was classified into 3 groups: responder (CST reduction of ≥ 20% from baseline and/or CST < 250 µm), stable (−20% < baseline CST < +20%) and non-responder (CST gain of ≥ +20%).

### 2.5. Statistical Analysis

For metric variables, mean and standard deviation (SD) are reported. For categorical variables, absolute frequencies and percentages based on the number of non-missing observations are provided. To analyze the percentage change in CST at the different follow-up visits (12-, 24-, 36- and 48-week timepoints) compared to baseline, a mixed model was calculated (R-function lmer, R-package lme4 version 1.1-37) [17], with the percentage change in CST as the dependent variable and follow-up visit as the independent factor. Patient and the nested factor eye within patient were included as random intercepts to account for intra-patient correlation. Derived from this model, marginal means with Bonferroni-adjusted 95% confidence limits and Bonferroni-adjusted *p*-values (H0: marginal mean equal to zero) were calculated for each follow-up visit (R-function emmeans, R-package emmeans version 1.11.2-8) [18].

To compare VA (approximate ETDRS letters) at follow-up visits with baseline, a mixed model was calculated as described above with VA (at baseline, 12-, 24-, 36- and 48-week timepoints) as the dependent variable. Each follow-up visit was then compared to baseline using Dunnett adjustment (R-function contrasts, R-package emmeans 1.11.2-8).

Response groups defined at the 12-week timepoint were descriptively compared by calculating the median (first quartile Q1; third quartile Q3) of CST and VA, as well as the percentage change in CST and the difference in VA compared to baseline for each subgroup. Graphical visualization of CST and VA was done by boxplots for response status at week 12 and each visit separately.

The two-sided significance level has been set to alpha = 0.05. The interpretation of the *p*-values is exploratory. Statistical analyses were conducted with R 4.5.1 [19].

### 2.6. Manuscript Preparation

The manuscript was drafted and revised by the authors based on the study findings and their interpretation. The authors were responsible for the scientific content and final version of the manuscript. During the preparation of this manuscript, the authors used ChatGPT (GPT 4.0; OpenAI; San Francisco, CA, USA) for the purposes of language and grammar refinement. The authors have reviewed and edited the output and take full responsibility for the content of this publication.

## 3. Results

### 3.1. Patient Population and Baseline Characteristics

Fifty-two eyes of 40 patients met the inclusion criteria. In Table 1, an overview of the baseline characteristics is presented.

Over the course of treatment, three patients were excluded from analysis. One because they underwent vitreoretinal surgery for vitreous hemorrhage during follow-up, and two because they were switched to another intravitreal agent due to insufficient treatment response.

### 3.2. Follow-Up

The mean ± SD follow-up time at each predefined timepoint was 14.53 ± 2.45 weeks at 12 weeks, 27.81 ± 2.68 weeks at 24 weeks, 41.16 ± 3.23 weeks at 36 weeks and 55.11 ± 5.87 weeks at 48 weeks. For clarity, we denote the timeframes as 12-, 24-, 36- and 48-week timepoints. The number of study eyes (patients) at the follow-up visits was 52 (40) at 12 weeks, 50 (38) at 24 weeks, 45 (34) at 36 weeks and 52 (40) at 48 weeks.

### 3.3. Morphology and Visual Acuity

At baseline, mean (±SD) VA was 66 ± 13.6 ETDRS letters and mean CST was 426.6 ± 106.7 µm.

The mean [Dunnett adjusted 95% confidence interval] VA change was +8.46 [4.89; 12.03] letters at 12 weeks (number of eyes (patients): n = 52 (40)), +7.57 (3.95; 11.18) letters at 24 weeks (n = 50 (38)), +7.65 (3.91; 11.39) letters at 36 weeks (n = 45 (34)) and +7.72 (3.80; 11.64) letters at 48 weeks (n = 39 (31)). The Dunnett-adjusted *p*-values for the VA change were *p* < 0.0001 at each visit.

The mean [Bonferroni-adjusted 95% confidence interval] percentage CST change was −28.05 [−36.27; −19.82] % at 12 weeks (n = 49 (38)), −27.01 [−35.20; −18.83] % at 24 weeks (n = 50 (38)), −29.46 [−37.89: −21.03] % at 36 weeks (n = 50 (38)) and −25.22 [−33.97; −16.48] % at 48 weeks (n = 39 (31)). The Bonferroni-adjusted *p*-values for the percentage CST changes from baseline were *p* < 0.0001 at each visit.

### 3.4. CST Response

At the 12-week visit (n = 49 eyes), 33 eyes (67%) were CST responders, 16 eyes (33%) remained stable, and 0 eyes were CST non-responders. At 24 weeks (n = 50 eyes), the results were 35 eyes (70%), 13 eyes (26%) and 2 eyes (4%) for the responder, stable and non-responder groups, respectively. At 36 weeks (n = 45), 36 eyes (80%) were responders, 8 eyes (18%) were stable, and 1 eye (2%) was a non-responder. After 48 weeks (n = 39 eyes), 25 eyes (64%) were responders, 13 (33%) had a stable CST, and 1 eye (3%) was a non-responder.

In Table 2, the individual CST response of each eye can be seen. Of the 33 eyes in the responder group at the 12-week visit, 14 eyes (42%) remained responders throughout all their visits after baseline. In addition, 10 eyes (30%) were responders at all their visits after baseline; however, each patient missed one visit. A total of 35 (67%) eyes were responders at their individual last documented visit; 6 eyes (12%) remained stable throughout the follow-up period, of which two eyes had one missing visit.

In terms of response status, 19 eyes had a heterogeneous follow-up course, switching once from responder to stable/non-responder (n = 7 eyes), from stable/non-responder to responder (n = 8 eyes) or changing their response status more than once (n = 4 eyes).

**Figure 1:**
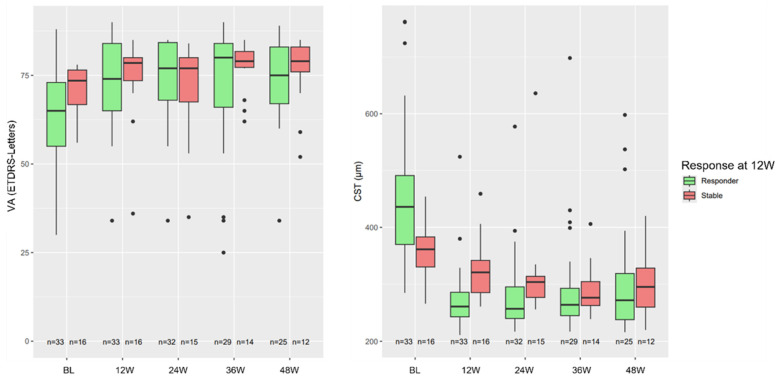
Boxplots of CST and VA at each visit grouped by their initial response at 12 weeks (BL = baseline, W = weeks).

In Figure 1, a comparison of the VA and CST of the responder and the stable group, classified by their initial response at 12 weeks, is shown. The two groups differ at baseline, with the responder group showing higher median CST (436 µm [responder] and 361.5 µm [stable]) and lower median VA (65.0 letters [responder] and 73.5 letters [stable]). These baseline differences diminished progressively, with minimal separation by weeks 36 and 48. The CST and VA results categorized by initial responder and stable groups are summarized in Table 3.

Across follow-up visits, median changes in CST and ETDRS visual acuity differed between responder and stable groups. For CST, responders showed a median reduction from baseline at 12 weeks (−34.3% [Q1 −49.1; Q3 −26.0]), which remained similar at subsequent visits (24 weeks: −35.7% [−46.9; −23.8]; 36 weeks: −37.0% [−46.3; −26.2]; 48 weeks: −29.7% [−47.8; −18.2]). In contrast, the stable group exhibited smaller median CST changes across visits (12 weeks: −12.5% [−15.0; −3.6]; 24 weeks: −15.5% [−20.6; −10.9]; 36 weeks: −20.6% [−26.7; −13.7]; 48 weeks: −17.3% [−26.9; −4.3]). Median VA changes from baseline in responders were positive at all follow-up visits, with a median gain of 9.0 letters at 12 weeks (Q1 2.0; Q3 14.0), remaining stable thereafter (24 weeks: 8.0 [1.75; 14.5]; 36 weeks: 9.0 [0.0; 16.0]; 48 weeks: 9.0 [0.0; 18.0]). The stable group showed smaller median VA changes, with gains of 4.5 letters at 12 weeks (Q1 1.75; Q3 7.0), 3.0 letters at 24 weeks (0.0; 6.5), 4.0 letters at 36 weeks (0.5; 7.75), and 6.0 letters at 48 weeks (2.25; 7.25).

### 3.5. Number of Injections

The mean number of injections administered during the monthly loading phase period was 2.56 ± 0.64. Over the first 6 months after baseline, patients received a mean of 4.02 ± 1.11 intravitreal faricimab injections (n = 52 eyes), while during the subsequent 6 months, the mean was 1.90 ± 0.98 injections (n = 42 eyes).

### 3.6. Treatment Interval

**Figure 2:**
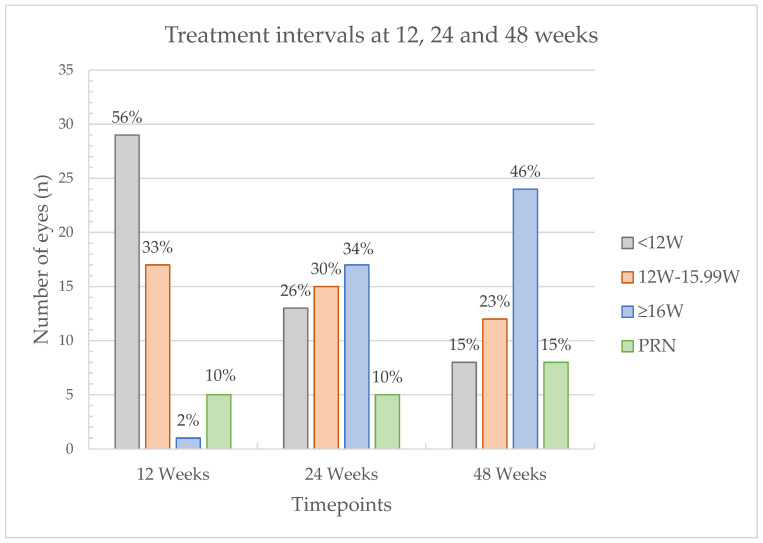
Bar graph of the frequency of individual treatment intervals at the 12-, 28- and 48-week timepoints. Sample sizes were 52, 50 and 52, at 14, 28 and 48 weeks, respectively.

Treatment intervals were extended at the discretion of the treating ophthalmologists in 4-week increments and, if necessary, reduced by 2- or 4-week increments. A minority of study eyes (5 out of 52 eyes, 10%) were started on a PRN regimen after the loading dose. At the primary endpoint of the study, 48 weeks after treatment initiation, 8 eyes (15%) were at a treatment interval of <12 weeks, 12 eyes (23%) were between 12 and 15.99 weeks, 24 eyes (46%) were at 16 weeks or longer, and 8 eyes (15%) were treated PRN. An overview of the frequency of treatment intervals is shown in Figure 2.

### 3.7. Intra- and Subretinal Fluid

At baseline (n = 52 eyes), all eyes (100%) exhibited intraretinal fluid (IRF) within the central 1 mm of the macula, and 21 eyes (40%) had subretinal fluid (SRF) in this region.

At follow-up, IRF and SRF within the central 1 mm were observed in 37 eyes (76%) and 3 eyes (6%) at week 12 (n = 49 eyes), in 31 eyes (62%) and 5 eyes (10%) at week 24 (n = 50 eyes), in 29 eyes (64%) and 2 eyes (4%) at week 36 (n = 45 eyes), and in 28 eyes (72%) and 4 eyes (10%) at week 48 (n = 39 eyes). However, the four eyes showing SRF at week 48 have all shown absence of SRF at previous follow-up timepoints.

## 4. Discussion

We evaluated the outcomes with faricimab treatment over 1 year in treatment-naïve eyes with diabetic macular edema.

In our study, faricimab was associated with clinically and statistically significant improvements in both CST and VA by week 12, and these gains were maintained throughout follow-up. Compared with the largest real-world treatment-naïve DME cohort to date (FARWIDE, 690 eyes), which reported a mean +4.7-letter gain at one year, our cohort achieved +7.7 letters at one year with comparable baseline VA (63.9 vs. 65.96 letters in our study) and a similar injection burden (months 1–6: 4.5 vs. 4.0; months 7–12: 1.9 vs. 1.9) [20].

Quah et al. documented an improvement from 61.1 ± 13.0 letters at baseline to 72.8 ± 11.5 letters by the fifth injection in their treatment-naïve cohort of 33 eyes [21]. In contrast, a single-center UK study (London, 34 treatment-naïve eyes) reported a non-significant VA change from 59.5 ± 13 letters at baseline to 62.7 ± 10.2 after 8 months despite substantial CST reduction [22]. Multicenter data from Japan (J-CREST, 67 treatment-naïve eyes) showed significant VA improvement from 0.341 ± 0.319 logMAR at baseline to 0.179 ± 0.279 logMAR at 6 months, directionally consistent with our one-year result [23]. A Spanish single-center series reported a mean improvement of +0.16 Snellen decimal after 8 months in 28 treatment-naïve eyes [24]. Taken together, these datasets position our cohort toward the upper range of real-world visual gains, with the discrepancy between the studies most likely reflecting their real-world nature.

At the primary endpoint, treatment interval at week 48, our results aligned with the results of the faricimab arms of YOSEMITE/RHINE, in which roughly 51–53% of eyes were at 16-week intervals and around 71–74% achieved intervals of 12 weeks or longer by year one [9]. In a real-world context, FARWIDE-DMO reported a mean number of injections of 4.5 in months 1–6 and 1.9 in months 7–12 for 690 treatment-naïve eyes [20]. Tsilegeridis-Legeris et al. reported that 66% of 63 treatment-naïve eyes achieved injection intervals of ≥12 weeks at one year, with a mean of 4.7 injections during the first six months and 2.2 injections during the second six months of the first treatment year [25]. The mean number of injections over the same periods in our study showed a very similar injection frequency. The number of faricimab injections in the loading phases ranged from one to four loading doses in DME real-world studies [20,22,23,26,27]. In our study, we reported a mean of 2.56 ± 0.64 injections until the 12-week follow-up, corresponding to the loading phase. All approaches have demonstrated significant visual and anatomical improvements with acceptable safety profiles. As far as real-world data is concerned, the loading phase as well as the subsequent treatment regimen remain highly individual.

There is no standardized definition for response to anti-VEGF therapy in DME. However, there have been approaches to define persistent DME based on anatomical response, with CST changes ranging from ≤10% to ≤25% being regarded as persistent DME [28,29,30,31,32,33]. We used the criteria, previously described by Huber et al., which defined a treatment response as either a ≥20% reduction in CST or a return of CST to normal limits (<250 µm) [11]. In our study, we descriptively analyzed VA and CST separately for the responder and stable groups formed by their initial CST response at 12 weeks.

The stable group showed lower baseline median CST (361.5 µm in the stable group vs. 436.0 µm in the responder group) and higher baseline median VA (73.5 stable group, 65.0 responder group). The pattern of improvement suggests that the apparent lack of response in the stable group may reflect ceiling effects—higher baseline VA and lower CST limiting measurable gains—rather than a lack of treatment effect. Large randomized trials (e.g., YOSEMITE and RHINE [9]) mitigate this issue with strict inclusion and exclusion criteria. In contrast, in our real-world study, eyes are included consecutively, irrespective of their baseline VA and CST, to assess effectiveness under routine care.

However, 10 of 16 eyes (62.5%) initially categorized as stable went on to be categorized as responders at a later follow-up time point (Table 2), suggesting that the lack of early response in treatment-naïve eyes may not exclude later morphological improvement. Moreover, CST changes in the stable group demonstrated continued improvement over follow-up, whereas CST changes in the responder group remained relatively stable between weeks 12 and 48. A similar pattern was observed for VA changes, with gradual gains over time in the stable group compared with plateauing improvements in responders. This aligns with DRCR Protocol T analyses showing that limited CST reduction at 12 weeks did not necessarily preclude meaningful long-term (2-year) vision gains with continued anti-VEGF therapy [6].

We analyzed the presence of intraretinal and subretinal fluid in the foveal area (central 1 mm) over the course of treatment. Foveal IRF presence decreased from 100% to 79%, while SRF fell from 40% to 10%, indicating a modest IRF and a pronounced SRF resolution over 48 weeks. However, every eye with remaining SRF at one year has shown SRF absence in a previous follow-up. In a YOSEMITE/RHINE post hoc analysis, anatomic outcomes in the central subfield were also tracked, including CST and absence of IRF/SRF. The majority of patients did not show any presence of SRF at week 16 (≈96–97% free from SRF), whereas IRF resolution progressed from 33–41% at year one to 44–49% at year two in the faricimab treat-and-extend arm [34]. Real-world data analyzing these fluid-compartment dynamics in treatment-naïve eyes with DME remain sparse. Quah et al. reported a reduction in treatment-naïve patients exhibiting IRF and SRF from 100% and 30.3% at baseline to 73.3% and 0.0% after five faricimab injections, respectively [21]. Murao et al. reported the absence of cystoid macular edema and SRF in 55% and 97% of treatment-naïve eyes after 6 months of faricimab treatment, respectively. Moreover, SRF reduction correlated with visual gains in treatment-naïve eyes [23].

Importantly, treatment durability in this study should not be interpreted solely on the basis of the achieved retreatment interval. Durability should also be considered in the context of sustained anatomical disease control over time. Accordingly, in addition to the treatment interval, we assessed anatomical response using CST response patterns and central OCT fluid status throughout follow-up. The finding that four eyes with previously resolved SRF showed recurrent SRF at 1 year, together with the observation that 19 eyes exhibited a heterogeneous CST response over the course of follow-up, underscores that disease recurrence remained a relevant challenge during interval extension. These findings suggest that longer treatment intervals do not necessarily equate to stable disease control in all eyes, and that apparent durability should be interpreted with caution when anatomical fluctuations persist. In this context, durability may be better understood as the ability to maintain both extended dosing intervals and consistent anatomical stability.

This study has several limitations. First, its single-center, retrospective design and modest sample size (52 eyes from 40 patients) limit generalizability. Second, post-loading management was determined at the discretion of the treating physician (T&E or PRN), which may confound treatment-interval estimates and hinder direct comparison with protocolized PTI regimens. Third, the absence of a comparator group prevents conclusions regarding comparative effectiveness or durability versus other anti-VEGF agents. Finally, the 48-week follow-up should be interpreted as first-year evidence and does not allow firm conclusions regarding long-term durability.

To conclude, in a real-world, treatment-naïve DME cohort, first-line faricimab yielded sustained functional and anatomical improvements over 48 weeks, together with meaningful treatment-interval extension. These findings, broadly consistent with clinical-trial and real-world data, support faricimab as an effective first-line option in routine practice while highlighting that the lack of early anatomical changes does not preclude later improvements under continued therapy. Prospective, multicenter studies with longer follow-up are warranted to confirm durability and define predictors of long-term extension.

## Figures and Tables

**Table 1:** Patient characteristics at baseline. Categorical variables are reported as counts (N), and metric variables are reported as mean ± standard deviation.

Baseline Characteristics	
Number of eyes/number of patients	52/40
Patient age (years)	62.23 ± 10.77
Gender (female/male)	19/21
Diabetes mellitus type (1/2)	4/36
Insulin-dependency (no/yes/missing)	16/8/16
Time since diabetes diagnosis (years)	17.59 ± 11.80, missing: 3
Last known HbA1c (%)	8.55 ± 2.04, missing: 7
Diabetic retinopathy (DR) stage (N)	
Mild non-proliferative DR (NPDR)	4
Moderate NPDR	18
Severe NPDR	24
Proliferative DR	4
Quiescent PDR	2
VA (ETDRS-Letters/LogMAR)	65.96 ± 13.55/0.38 ± 0.27
Mean CST (µm)	426.56 ± 106.72
Prior ocular Surgery (multiple categories per case possible)	
Panretinal laser coagulation/missing	2/2
Macular laser coagulation/missing	0/2
Cataract surgery/missing	10/1
Vitrectomy/missing	0/2

**Table 2:** Individual CST response at each follow-up visit (NA = no data available). For easier identification of each individual response status, the categories are additionally color-coded in the background: NA = grey, responder = green, stable = yellow, non-responder = orange.

Week 12	Week 24	Week 36	Week 48	N
NA	Responder	NA	NA	1
NA	Responder	Responder	Responder	1
NA	Responder	Responder	Stable	1
Responder	NA	Responder	Responder	1
Responder	Non-responder	Responder	Responder	1
Responder	Responder	NA	Responder	2
Responder	Responder	NA	Stable	1
Responder	Responder	Non-responder	Non-responder	1
Responder	Responder	Responder	NA	7
Responder	Responder	Responder	Responder	14
Responder	Responder	Responder	Stable	2
Responder	Responder	Stable	Stable	1
Responder	Stable	NA	Stable	1
Responder	Stable	Responder	Stable	1
Responder	Stable	Stable	NA	1
Stable	NA	Responder	NA	1
Stable	Non-responder	Responder	Responder	1
Stable	Responder	NA	Responder	1
Stable	Responder	Responder	NA	1
Stable	Responder	Responder	Responder	2
Stable	Stable	NA	Responder	1
Stable	Stable	Responder	Responder	1
Stable	Stable	Responder	Stable	2
Stable	Stable	Stable	NA	2
Stable	Stable	Stable	Stable	4

**Table 3:** Median CST (µm) and VA (ETDRS Letters) of the initial (12-week visit) responder and stable groups and of the overall cohort at each follow-up visit (BL = Baseline; W = week).

Visit	Median CST (Q1; Q3)			Median VA (Q1; Q3)			N (r/s/o)
	Responder (r)	Stable (s)	Overall (o)	Responder (r)	Stable (s)	Overall (o)	
BL	436.0 (370.0; 491.0)	361.5 (330.5; 383.25)	393.5 (353.75; 475)	65.0 (55.0; 73.0)	73.5 (66.75; 76.5)	69.0 (60.0; 75.25)	33/16/52
W 12	261.0 (243.0; 286.0)	321.0 (285.5; 342.0)	278.0 (253.0; 311.0)	74.0 (65.0; 84.0)	78.5 (73.5; 80.0)	77.5 (71.75; 82.25)	33/16/52
W 24	257.0 (240.0; 295.5.0)	304.0 (277.0; 314.0)	266.5 (243.75; 308.25)	77.0 (68.0; 84.25)	77.0 (67.5; 80.0)	77.5 (69.25; 82.75)	32/15/50
W 36	264.0 (245.0; 293.0)	276.5 (262.75; 304.75)	269.0 (247.0; 308.0)	80.0 (66.0; 84.0)	79.0 (77.25; 81.75)	80.0 (68.0; 83.0)	29/14/45
W 48	272.0 (238.0; 319.0)	295.5 (260.0; 328.5)	278.0 (249.0; 326.5)	75.0 (67.0; 83.0)	79.0 (76.0; 83.0)	77.0 (68.0; 83.0)	25/12/39

## Data Availability

The datasets analyzed during the current study are not publicly available due to the inclusion of sensitive patient data and applicable data protection regulations. The data are available from the corresponding author on reasonable request, subject to approval by the relevant institutional review board and in accordance with applicable data protection and ethical guidelines.

## References

[1] Cheung N., Mitchell P., Wong T.Y. (2010). Diabetic retinopathy. Lancet.

[2] Kusuhara S., Fukushima Y., Ogura S., Inoue N., Uemura A. (2018). Pathophysiology of Diabetic Retinopathy: The Old and the New. Diabetes Metab. J..

[3] Nguyen Q.D., Brown D.M., Marcus D.M., Boyer D.S., Patel S., Feiner L., Gibson A., Sy J., Rundle A.C., Hopkins J.J. (2012). Ranibizumab for diabetic macular edema: Results from 2 phase III randomized trials: RISE and RIDE. Ophthalmology.

[4] Ashraf M., Souka A., Adelman R. (2016). Predicting outcomes to anti-vascular endothelial growth factor (VEGF) therapy in diabetic macular oedema: A review of the literature. Br. J. Ophthalmol..

[5] Korobelnik J.F., Do D.V., Schmidt-Erfurth U., Boyer D.S., Holz F.G., Heier J.S., Midena E., Kaiser P.K., Terasaki H., Marcus D.M. (2014). Intravitreal aflibercept for diabetic macular edema. Ophthalmology.

[6] Bressler N.M., Beaulieu W.T., Maguire M.G., Glassman A.R., Blinder K.J., Bressler S.B., Gonzalez V.H., Jampol L.M., Melia M., Sun J.K. (2018). Early Response to Anti-Vascular Endothelial Growth Factor and Two-Year Outcomes Among Eyes With Diabetic Macular Edema in Protocol T. Am. J. Ophthalmol..

[7] Wells J.A., Glassman A.R., Ayala A.R., Jampol L.M., Bressler N.M., Bressler S.B., Brucker A.J., Ferris F.L., Hampton G.R., Jhaveri C. (2016). Aflibercept, Bevacizumab, or Ranibizumab for Diabetic Macular Edema: Two-Year Results from a Comparative Effectiveness Randomized Clinical Trial. Ophthalmology.

[8] Sahni J., Patel S.S., Dugel P.U., Khanani A.M., Jhaveri C.D., Wykoff C.C., Hershberger V.S., Pauly-Evers M., Sadikhov S., Szczesny P. (2019). Simultaneous Inhibition of Angiopoietin-2 and Vascular Endothelial Growth Factor-A with Faricimab in Diabetic Macular Edema: BOULEVARD Phase 2 Randomized Trial. Ophthalmology.

[9] Wykoff C.C., Abreu F., Adamis A.P., Basu K., Eichenbaum D.A., Haskova Z., Lin H., Loewenstein A., Mohan S., Pearce I.A. (2022). Efficacy, durability, and safety of intravitreal faricimab with extended dosing up to every 16 weeks in patients with diabetic macular oedema (YOSEMITE and RHINE): Two randomised, double-masked, phase 3 trials. Lancet.

[10] Glassman A.R., Wells J.A., Josic K., Maguire M.G., Antoszyk A.N., Baker C., Beaulieu W.T., Elman M.J., Jampol L.M., Sun J.K. (2020). Five-Year Outcomes after Initial Aflibercept, Bevacizumab, or Ranibizumab Treatment for Diabetic Macular Edema (Protocol T Extension Study). Ophthalmology.

[11] Huber K.L., Stino H., Steiner I., Fuchs P., Goldbach F., Mai J., Gerendas B.S., Kriechbaum K., Schmidt-Erfurth U., Pollreisz A. (2024). Real-World Outcomes After Switch From Aflibercept to Faricimab in Eyes With Diabetic Macular Edema. Investig. Ophthalmol. Vis. Sci..

[12] Savant S.V., Kwan J.T., Barouch F., Chang J., Ramsey D.J., Marx J., Blaha G., Klein-Mascia K. (2024). Durability and Efficacy of Faricimab in Treatment-Resistant Retinal Edema Utilizing “Real-World” Dosing Regimens. J. Ophthalmol..

[13] Chakraborty D., Das S., Maiti A., Sinha T., Das A., Sheth J., Boral S., Mondal S., Nandi K. (2025). Clinical Evaluation of Faricimab in Real-World Diabetic Macular Edema in India- A Multicenter Observational Study. Clin. Ophthalmol..

[14] Al-Rufayie M., Palmieri F., Hamoud Bedan A., Younis S., Ali A., Kurumthottical M., Taechameekietichai T., Fabozzi L. (2024). Real-World Results in Treating Diabetic Macular Edema With Faricimab at a London-Based Tertiary Eye Hospital. Cureus.

[15] Hirakata T., Hara F., Nochi Y., Shinohara D., Yamamoto S., Hiratsuka Y., Nakao S. (2025). Short-term real-world outcomes of diabetic macular edema treated with intravitreal faricimab. PLoS ONE.

[16] Gregori N.Z., Feuer W., Rosenfeld P.J. (2010). Novel method for analyzing snellen visual acuity measurements. Retina.

[17] Bates D., Mächler M., Bolker B., Walker S. (2015). Fitting Linear Mixed-Effects Models Using lme4. J. Stat. Softw..

[18] Piaskowski R.V.L.J. (2025). Emmeans: Estimated Marginal Means, Aka Least-Squares Means.

[19] Team R.C. (2021). R: A Language and Environment for Statistical Computing.

[20] Peto T., Pearce I., Talks J., de Salvo G., Patel P.J., de Silva S.R., Gale R.P., Sivaprasad S., Varma D., Reynolds R. (2025). Real-world treatment patterns and visual outcomes of faricimab in patients with diabetic macular oedema in the UK at 12 months: The FARWIDE-DMO study. Eye.

[21] Quah N.Q.X., Javed K., Arbi L., Hanumunthadu D. (2024). Real-World Outcomes of Faricimab Treatment for Neovascular Age-Related Macular Degeneration and Diabetic Macular Edema. Clin. Ophthalmol..

[22] Khetan T., Shah P., Ahmed S., Jeyabaladevan P., Vithlani S. (2025). Real-world outcomes of faricimab in treating diabetic macular edema up to two years. Indian J. Ophthalmol..

[23] Murao F., Yanai R., Kusuhara S., Abe Y., Shimura M., Ohara H., Terasaki H., Sugimoto M., Matsubara H., Hirano Y. (2025). Visual outcomes and anatomical biomarkers of Faricimab for diabetic macular edema in the J-CREST real-world comparison of naïve and treated eyes. Sci. Rep..

[24] Esteban-Floría O., Mateo J., Lara J., Bartolomé I., Herrero I., Pérez M.A., Cabello C., Honrubia A., Pinilla I., Ascaso J. (2025). Efficacy and Safety of Faricimab in Diabetic Macular Edema: Real-World Outcomes in Treatment-Naïve and Previously Treated Eyes. J. Clin. Med..

[25] Tsilegeridis-Legeris T., Kenawy N. (2025). Beyond the First Year of Intravitreal Faricimab for Diabetic Macular Oedema: A Single-Centre Experience. Cureus.

[26] Mizuki Y., Inokuchi S., Kuroki T., Kamata A., Onishi J., Watanabe Y., Takeda M., Meguro A., Kawagoe T., Teshigawara T. (2025). Two-Year Real-World Outcomes of Faricimab for Diabetic Macular Edema in Japan. Clin. Ophthalmol..

[27] Śpiewak D., Drzyzga Ł., Dorecka M., Witek K., Wyględowska-Promieńska D. (2025). Efficacy of Faricimab in the Treatment of Diabetic Macular Edema and Faricimab-Related Changes in OCT and OCT Angiography. Pharmaceutics.

[28] Busch C., Zur D., Fraser-Bell S., Laíns I., Santos A.R., Lupidi M., Cagini C., Gabrielle P.H., Couturier A., Mané-Tauty V. (2018). Shall we stay, or shall we switch? Continued anti-VEGF therapy versus early switch to dexamethasone implant in refractory diabetic macular edema. Acta Diabetol..

[29] Dugel P.U., Campbell J.H., Kiss S., Loewenstein A., Shih V., Xu X., Holekamp N.M., Augustin A.J., Ho A.C., Gonzalez V.H. (2019). ASSOCIATION BETWEEN EARLY ANATOMIC RESPONSE TO ANTI-VASCULAR ENDOTHELIAL GROWTH FACTOR THERAPY AND LONG-TERM OUTCOME IN DIABETIC MACULAR EDEMA: An Independent Analysis of Protocol i Study Data. Retina.

[30] Klein K.A., Cleary T.S., Reichel E. (2017). Effect of intravitreal aflibercept on recalcitrant diabetic macular edema. Int. J. Retin. Vitr..

[31] Koyanagi Y., Yoshida S., Kobayashi Y., Kubo Y., Nakama T., Ishikawa K., Nakao S., Hisatomi T., Ikeda Y., Oshima Y. (2018). Visual Outcomes Based on Early Response to Anti-Vascular Endothelial Growth Factor Treatment for Diabetic Macular Edema. Ophthalmologica.

[32] Shah A.R., Yonekawa Y., Todorich B., Van Laere L., Hussain R., Woodward M.A., Abbey A.M., Wolfe J.D. (2017). Prediction of Anti-VEGF Response in Diabetic Macular Edema After 1 Injection. J. Vitreoretin. Dis..

[33] Sorour O.A., Levine E.S., Baumal C.R., Elnahry A.G., Braun P., Girgis J., Waheed N.K. (2023). Persistent diabetic macular edema: Definition, incidence, biomarkers, and treatment methods. Surv. Ophthalmol..

[34] Lim J.I., Amador M.J., Dhoot D.S., Finn A., Fraser-Bell S., Gibson K., Idowu O.O., Khurana R.N., Lanzetta P., Lin T.C. (2025). Anatomic Control with Faricimab versus Aflibercept in the YOSEMITE/RHINE Trials in Diabetic Macular Edema. Ophthalmol. Retin..

